# Supplementary material for: Highly Luminescent Crystalline Sponge: Sensing Properties and Direct X-ray Visualization of the Substrates
Source: Molecules. 2022 Nov 19;27(22):8055. doi: 10.3390/molecules27228055 (PMC9692560; doi:10.3390/molecules27228055)
Supplement: Supplementary file 1 [file molecules-27-08055-s001.zip › molecules-2010905-supplementary.pdf]

Supporting information

Highly Luminescent Crystalline Sponge: Sensing Properties and Direct X-ray Visualization of the Substrates

Pavel A. Demakov*, Alexey A. Ryadun, Danil N. Dybtsev

Nikolaev Institute of Inorganic Chemistry, Siberian Branch of the Russian Academy of Sciences, 3 Akad. Lavrentieva Ave., Novosibirsk 630090, Russia
(A.A.R.) Correspondence: demakov@niic.nsc.ru

Table S1. Bond lengths in Eu^{3+} coordination environment

Part of the capped square antiprism		Structure				
	O atoms	$\mathbf{1}_{DMF}$ 140 K [MS34]	$\mathbf{1}_{DMF}$ RT	$\mathbf{1}_{phet}$	$\mathbf{1}_{cin}$	$\mathbf{1}_{DMSO}$
Base square	Solvent	2.400(5)<br>2.407(5)	2.414(3)<br>2.417(3)	2.419(2)<br>2.422(2)	2.414(5) –<br>2.445(5)	2.392(4)<br>2.405(4)
	κ^2-COO	2.459(4)<br>2.489(4)	2.470(3)<br>2.487(3)	2.433(2)<br>2.503(2)	2.475(4) –<br>2.488(4)	2.464(4)<br>2.506(4)
Upper square	κ^1,κ^1-COO	2.388(4)<br>2.427(4)	2.384(3)<br>2.412(3)	2.423(2)<br>2.430(2)	2.375(4) –<br>2.418(4)	2.388(4)<br>2.429(3)
	κ^2,κ^1-COO	2.355(4)<br>2.450(4)	2.351(3)<br>2.454(3)	2.365(2)<br>2.473(2)	2.338(4) –<br>2.459(4)	2.350(4)<br>2.462(4)
Upper square cap		2.675(4)	2.715(3)	2.624(2)	2.690(4),<br>2.692(4)	2.806(4)

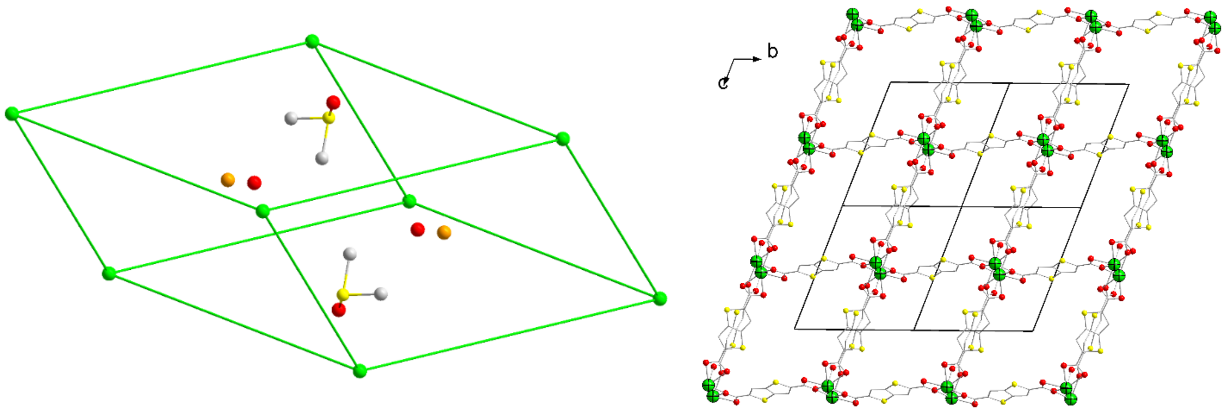

a b

Figure S1. Localization of guest molecules in **1**$_{DMSO}$. Each binuclear Eu(III)-carboxylate block is shown as a node, each ttdc linker is shown as an edge. Disordered water positions are shown in different shades (**a**). View of Eu-ttdc coordination lattice along *a* axis in **1**$_{DMSO}$ (**b**). Only one of two possible positions for the disordered thienothiophene moietties are shown for clarity.

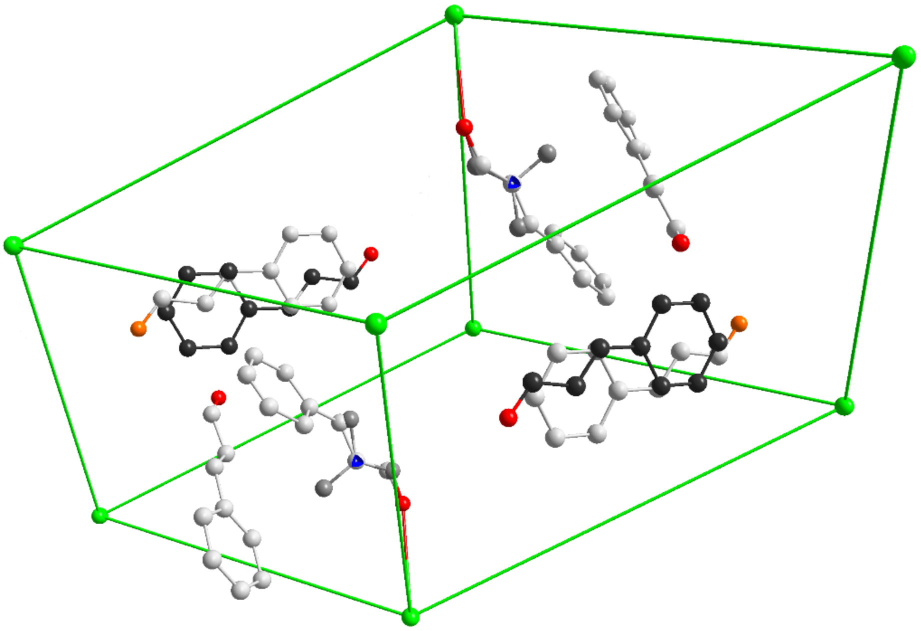

Figure S2. Localization of cinnamal molecules in **1**$_{cin}$. Eu-ttdc coordination lattice is shown accordingly to the Fig S1. Disordered moieties are shown in different shades.

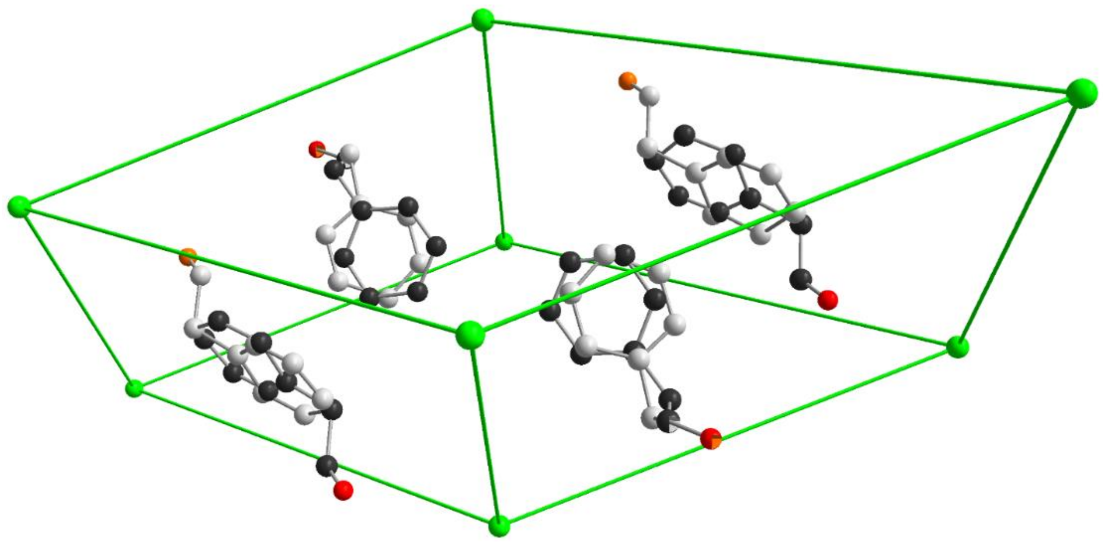

Figure S3. Localization of phenylethanal molecules in $\mathbf{1}_{phet}$. Eu-ttdc coordination lattice is shown accordingly to the Fig S1. Disordered moieties are shown in different shades.

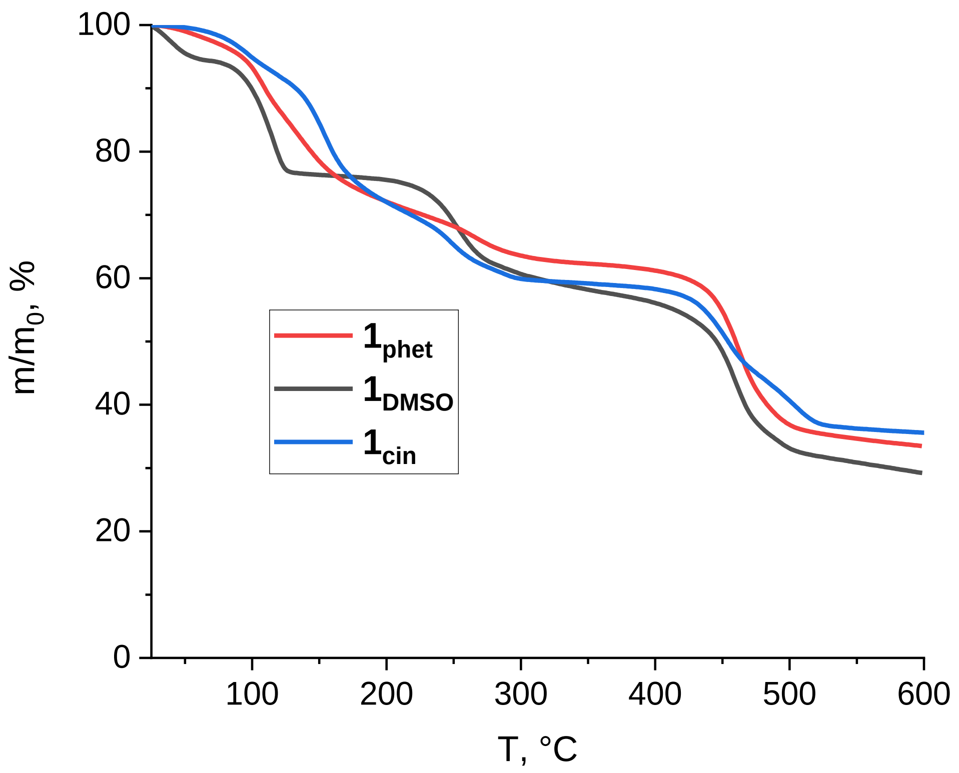

Figure S4. TG plots for the adducts.

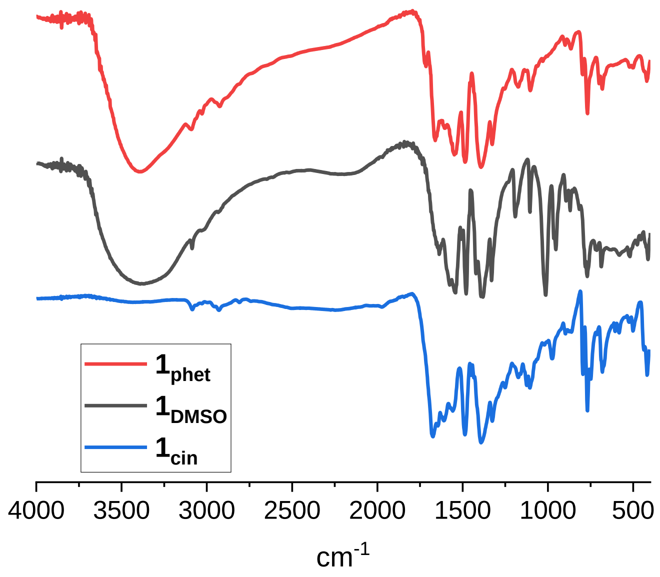

Figure S5. Infrared spectra of the adducts.

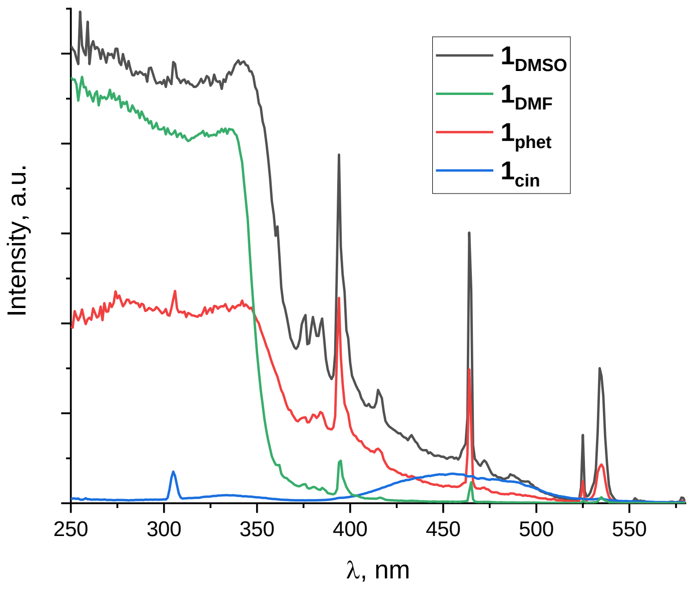

Figure S6. Excitation spectra for $\mathbf{1}_{solv}$ at λ_{em} = 613 nm.

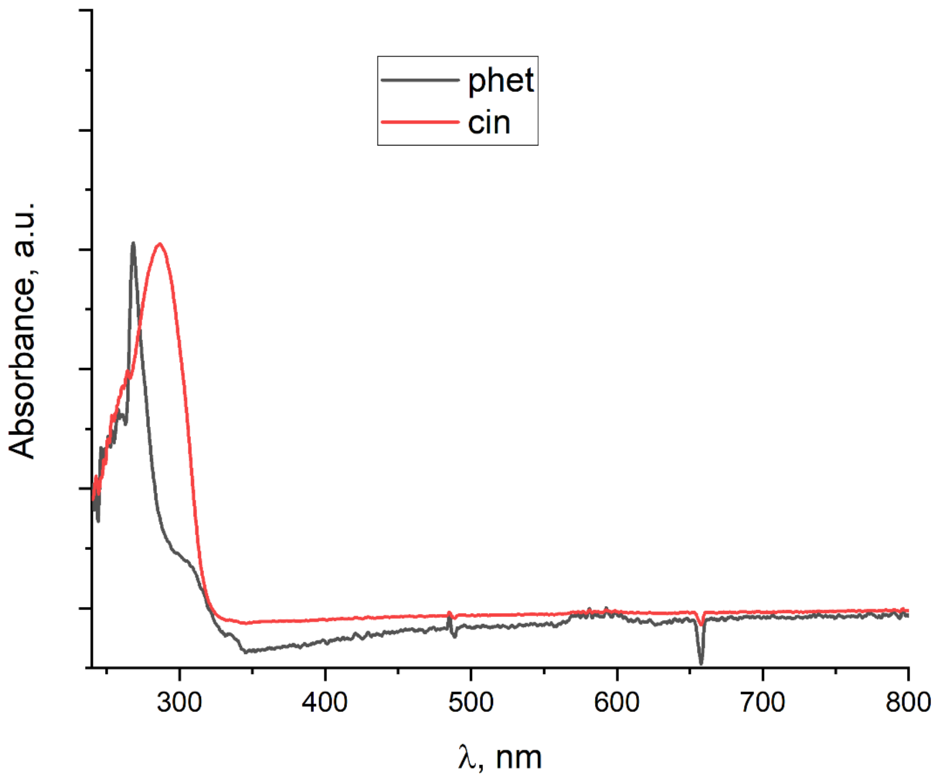

Figure S7. Normalized absorption spectra of cinnamal and phenylethanal (10^{-4} M) in DMF solutions.

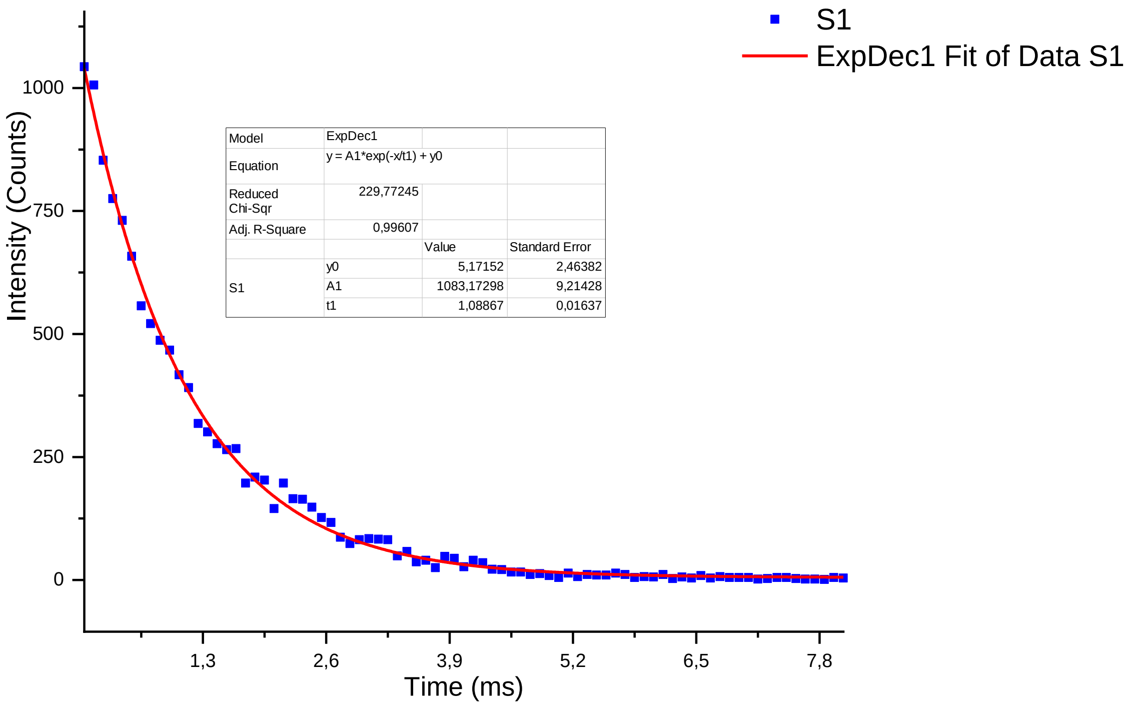

Figure S8. Decay curve for the emission of **1**$_{DMF}$ in solid state.

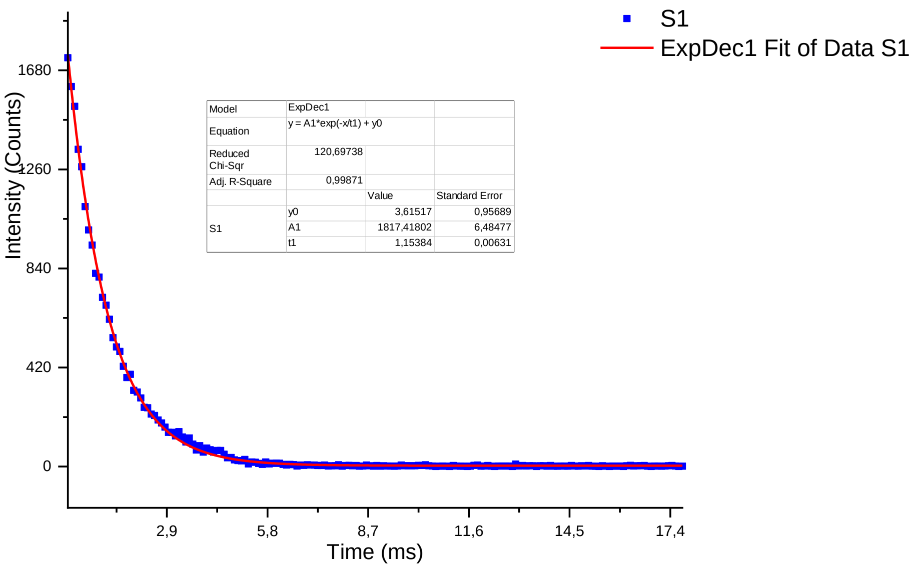

Figure S9. Decay curve for the emission of **1**$_{DMSO}$ in solid state.

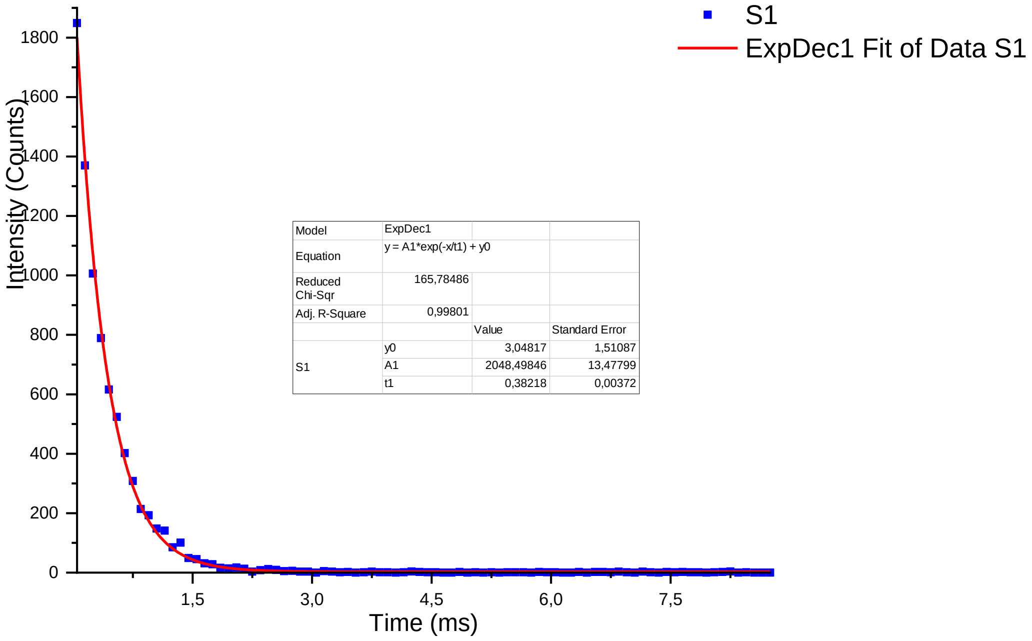

Figure S10. Decay curve for the emission of **1**$_{\mathbf{phet}}$ in solid state.

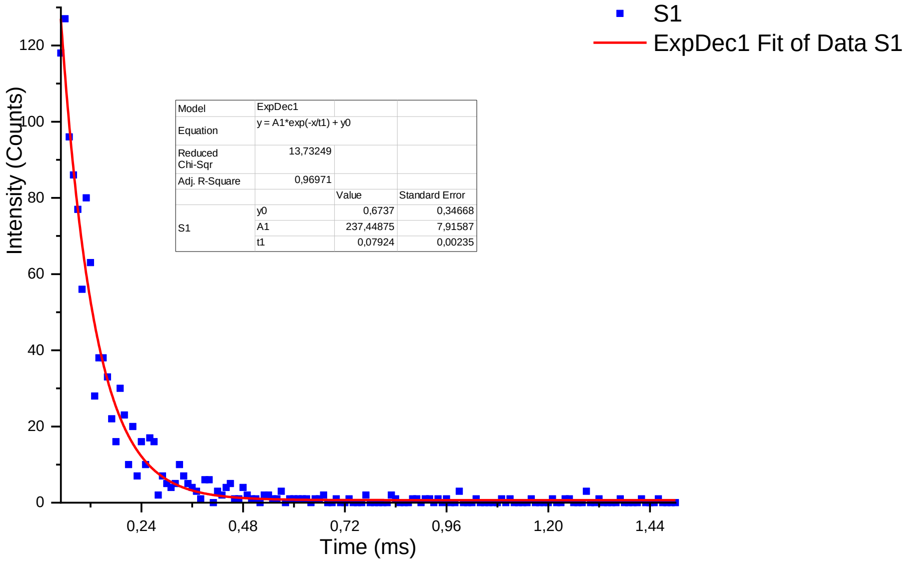

Figure S11. Decay curve for the emission of **1**$_{\mathbf{cin}}$ in solid state.

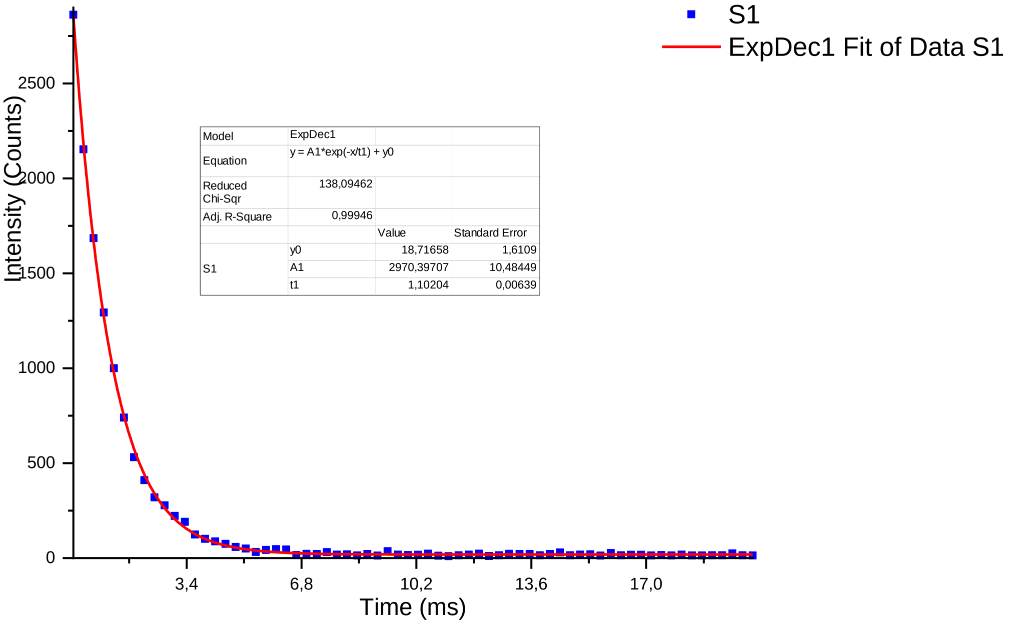

Figure S12. Decay curve for the emission of $\mathbf{1}_{\mathrm{DMF}}$ suspended in DMF.

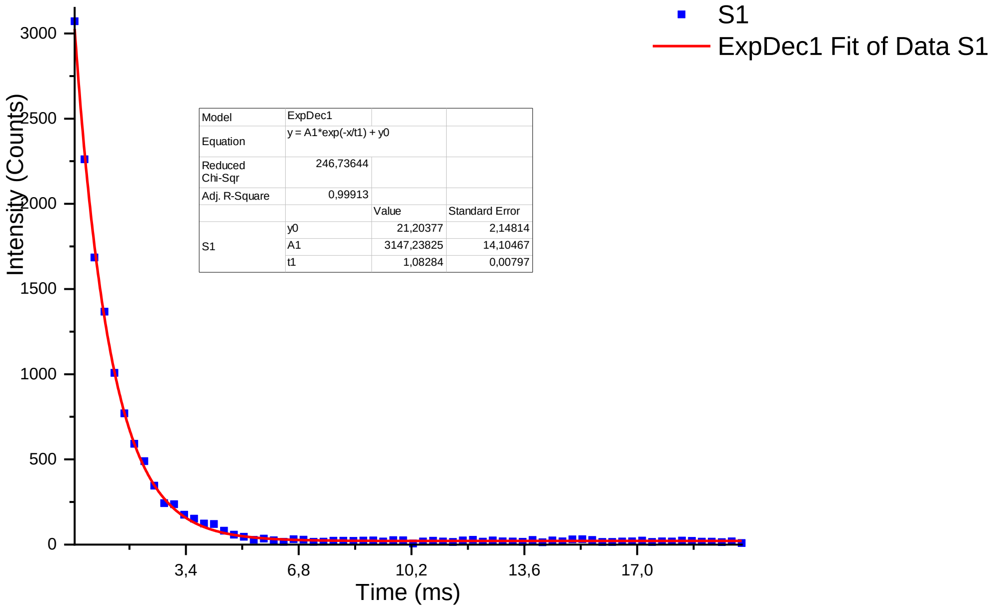

Figure S13. Decay curve for the emission of $\mathbf{1}_{\mathrm{DMF}}$ suspended in 1% solution of DMSO in DMF.

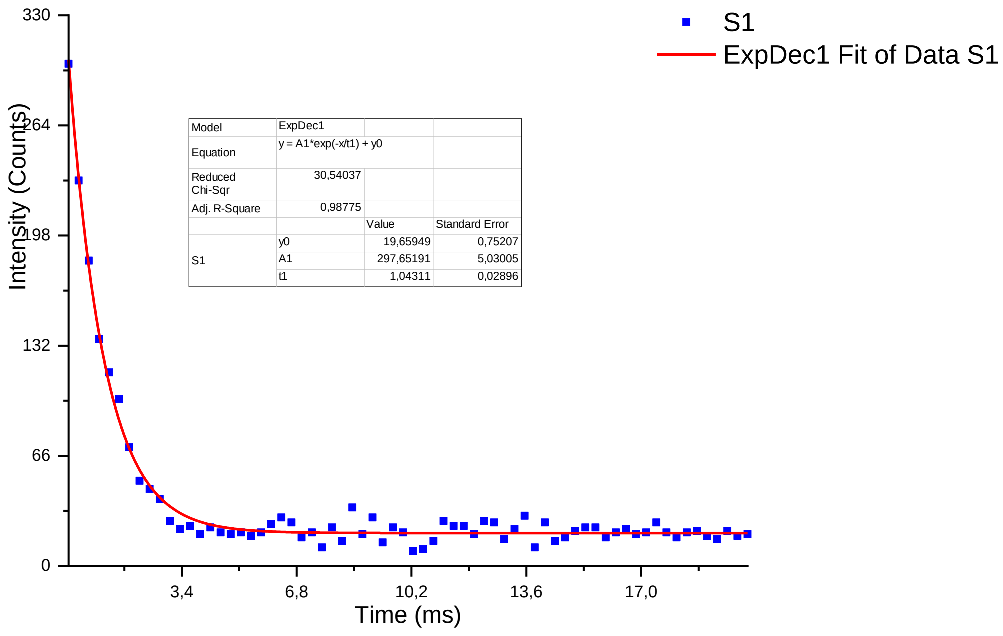

Figure S14. Decay curve for the emission of **1**DMF suspended in 1% solution of phenylethanal in DMF.

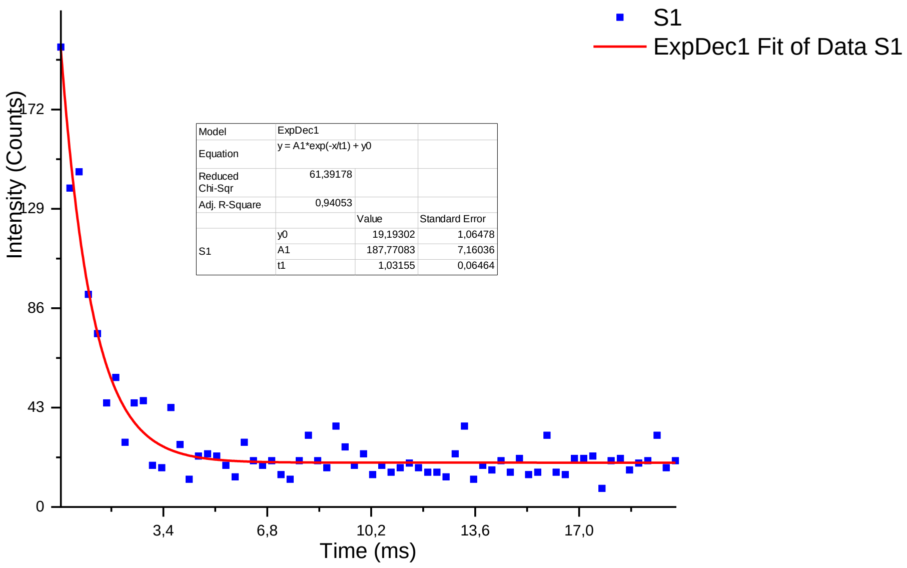

Figure S15. Decay curve for the emission of **1**DMF suspended in 1% solution of cinnamal in DMF.
